# More than Attendance Frequencies: A Deeper Look at Religion, Spirituality, and Aging Research

**DOI:** 10.1093/geroni/igaf122.1046

**Published:** 2025-12-31

**Authors:** Nirmala Lekhak, Stephen Fogle

## Abstract

Recent decades of gerontological research in religion, spirituality, and aging have produced innovative approaches toward understanding the depth and diversity of older adults’ lived experiences. The Religion, Spirituality, and Aging Interest Group annual symposia frequently provides a forum for dynamic conversations regarding the state of research including attention to “The Next Generation” (2019), “Forgotten Variables” (2020), “Spiritual Care” (2021) and, “Spiritual Needs” (2023). Continuing this perennial conversation, this symposium presents original contributions to provide a deeper look at emergent methods and variables of interest guiding contemporary conception and operationalization of religion and spirituality (R/S) in gerontological research. The first paper will present an examination of the extent of engagement in R/S among older adults hospitalized for acute coronary syndrome (ACS) and their association with physical health related quality of life (HRQoL). Second paper explores how death conversations and experiences contribute to one’s understanding of death, including the influence of religious vocabulary, funerary customs, and after-life beliefs. Third paper offers a scoping review to understand religion and spirituality variables as found in population health data commonly used for older adult research. The last paper is a qualitative study focused on how current and former dementia caregivers perceive effects of Maitri Sambodh Dhyaan (MSD) meditation. After attending this symposium, participants will be able to 1) demonstrate knowledge of the current state of religion, spirituality, and aging research; 2) identify specific variables related to religious and spiritual practices and experiences; 3) connect emergent religion, spirituality, and aging research to health and well-being outcomes. Religion, Spirituality and Aging Interest Group Sponsored Symposium

